# Effect of positive end-expiratory pressure on right ventricle function assessed by speckle tracking echocardiography

**DOI:** 10.1186/cc13471

**Published:** 2014-03-17

**Authors:** SR Orde, A Behfar, PG Stalboerger, JK Oh, GC Kane

**Affiliations:** 1 Mayo Clinic, Rochester, MN, USA

## Introduction

We sought to determine in a swine model whether a novel echocardiography method, speckle tracking echocardiography (STE), could determine deterioration in right ventricle (RV) function induced by escalating levels of positive end-expiratory pressure (PEEP), and to compare STE with RV fractional area change (FAC). Acute cor pulmonale and hypotension can be induced by high levels of PEEP used in the management of acute respiratory distress syndrome [1]. Quantifying RV function by echocardiography can be challenging due to its shape and position. STE is a relatively new, feasible, sensitive, angle-independent method for describing cardiac deformation (strain) [2] and is particularly useful in analyzing RV function (RV free wall strain, RVfwS), as has been shown in pulmonary hypertension cohorts [3].

## Methods

Ten pigs, 40 to 90 kg, anaesthetized, fully mechanically ventilated at 6 to 8 mg/kg were subject to stepwise escalating levels of PEEP at 2-minute intervals (0, 5, 10, 15, 20, 25 and 30 cmH_2_O). RV images were obtained using intracardiac echocardiography (for optimal frame- rate and endocardial definition) and were analyzed offline for FAC and RVfwS (using Velocity Vector Imaging; Siemens).

## Results

Escalating levels of PEEP were strongly associated with significant reductions in mean blood pressure (*R*^2 ^= 0.8, *P *< 0.0001), FAC (*R*^2 ^= 0.8, *P *< 0.0001) and RVfwS (*R*^2 ^= 0.9, *P *< 0.001). Paired t tests indicated significant reductions in RVfwS with each step increase in PEEP. FAC only showed significant deterioration at 15 cmH_2_O PEEP. Significant hypotension (a decrease of more than 20 mmHg) occurred after 10 cmH_2_O PEEP. RVfwS decreased by a larger extent and earlier than FAC and mean blood pressure with increasing levels of PEEP.

## Conclusion

RVfwS measured by STE is a sensitive method for determining RV dysfunction induced by PEEP. RVfwS displays a stronger association, greater deterioration and earlier reduction than FAC and mean blood pressure with escalating levels of PEEP. This potentially has interesting implications for the role of STE in managing PEEP levels in critically ill patients with acute lung injury.
